# Comparative Analysis on Vestibular Schwannoma Surgery with and without Intraoperative Fluorescein Sodium Enhancement

**DOI:** 10.3390/brainsci14060571

**Published:** 2024-06-03

**Authors:** Amer A. Alomari, Sadeen Sameer Eid, Flavia Fraschetti, Silvia Michelini, Luciano Mastronardi

**Affiliations:** 1 Department of Neurosurgery, San Filippo Neri Hospital/ASLRoma1, 00135 Rome, Italy; aaalomari07@med.just.edu.jo (A.A.A.); flavia.fraschetti@gmail.com (F.F.); 2 Division of Neurosurgery, Department of Special Surgery, Faculty of Medicine, Mutah University, Al-Karak 61710, Jordan; 3 Faculty of Medicine, Jordan University of Science and Technology, Irbid 22110, Jordan; sseid19@med.just.edu.jo; 4 Department of Neurosurgery, University of Tor Vergata, 00133 Rome, Italy; silviamichelini1992@gmail.com

**Keywords:** vestibular schwannoma, acoustic neuroma, fluorescein sodium, facial nerve function

## Abstract

Background: Vestibular schwannoma (VS), also known as acoustic neuroma, is a benign, well-encapsulated, and slow-growing tumor that originates from Schwann cells, which form the myelin sheath around the vestibulocochlear nerve (VIII cranial nerve). The surgical treatment of this condition presents a challenging task for surgeons, as the tumor’s location and size make it difficult to remove without causing damage to the surrounding structures. In recent years, fluorescein sodium (FS) has been proposed as a tool to enhance surgical outcomes in VS surgery. This essay will provide an analytical comparison of the use of FS in VS surgery, evaluating its benefits and limitations and comparing surgical outcomes with and without FS-assisted surgery. Methods: In a retrospective study conducted at San Filippo Neri Hospital, we examined VS cases that were operated on between January 2017 and December 2023. The patients were divided into two groups: group A, which consisted of patients who underwent surgery without the use of FS until January 2020 (102 cases), and group B, which included patients who underwent surgery with FS after January 2020 (55 cases). All operations were performed using the retrosigmoid approach, and tumor size was classified according to the Koos, et al. classification system. The extent of surgical removal was evaluated using both the intraoperative surgeon’s opinion and postoperative MRI imaging. Preoperatively and postoperatively, facial nerve function and hearing were assessed. In group B, FS was used to assist the surgical procedures, which were performed using a surgical microscope equipped with an integrated fluorescein filter. Postoperative clinical and MRI controls were performed at six months and annually, with no patients lost to follow-up. Results: This study investigated the impact of intraoperative fluorescein exposure on tumor resection and clinical outcomes in patients with VS. The study found a statistically significant difference in the tumor resection rates between patients who received fluorescein intraoperatively (*p* = 0.037). Further analyses using the Koos classification system revealed a significant effect of fluorescein exposure, particularly in the Koos 3 subgroup (*p* = 0.001). Notably, no significant differences were observed in hearing loss or facial nerve function between the two groups. A Spearman correlation analysis revealed a positive correlation between tumor size and Koos, age, and size, but no significant correlation was found between facial nerve function tests. Conclusions: FS-assisted surgery for VS may potentially enhance tumor resection, allowing for more comprehensive tumor removal.

## 1. Introduction

According to the World Health Organization (WHO), schwannomas are classified as grade I benign tumors, and they typically occur as solitary tumors in approximately 90% of cases. However, in some instances, multiple tumors can develop in the same individual, which can lead to syndromic associations such as neurofibromatosis type 2 (NF2), schwannomatosis, and Carney complex [1,2,3]. Among all nerve sheath tumors, schwannomas are the most common, with approximately 89% of them originating from the vestibular nerve. Notably, about 60% of all schwannomas are actually vestibular schwannomas (VSs) [4].

VS, also known as acoustic neuroma, is a benign, encapsulated, and slow-growing tumor that originates from Schwann cells, which form the myelin sheath around the vestibulocochlear nerve (VIII cranial nerve). These tumors account for approximately 8% of all intracranial tumors, and most patients diagnosed are in their fourth and sixth decades of life. The condition affects both men and women equally [5,6]. A patient’s clinical presentation may include symptoms such as reduced hearing, tinnitus, and imbalance, as well as facial nerve palsy, which is rare and may occur later in the course of the disease [7].

The treatment for VS involves a personalized approach, with the ultimate goal of addressing the unique needs and circumstances of each patient [8]. For small, asymptomatic schwannomas, a conservative approach involving observation with serial imaging and surgery only when the tumor shows growth may be the most appropriate option [9]. Conversely, surgery may be considered if the lesion is growing or causing symptoms. The decision on the treatment strategy depends on several factors, including the patient’s age, health history, hearing status, tumor size, and presence of NF2 [8].

Fluorescein sodium (FS) is a fluorescent dye that has been employed in various medical procedures, including ophthalmology, neurosurgery, and cardiology [10,11]. In VS surgery, FS is administered intravenously before surgery and accumulates in the tumor’s blood vessels. Under a specialized microscope filter (yellow 560), the tumor’s blood vessels glow, allowing the surgeon to differentiate between the tumor and surrounding structures and remove the tumor with greater precision [12].

Although there is a lack of research on the effects of FS in VS resection surgery, this study provides an opportunity to investigate this research gap. The primary objective of this study was to compare the surgical outcomes of FS-assisted surgery (FSAS) and non-FS-assisted surgery in terms of the extent of removal, facial nerve function, and hearing levels. By filling this knowledge gap, our study aims to provide important insights into the benefits of FSAS in achieving successful VS operations and address the safety profile and dosage for the use of intravenous FS in neurosurgical practice for VS surgery.

## 2. Materials and Methods

### 2.1. Study Design

We retrospectively reviewed all VS cases that underwent surgery by the last author (LM) at San Filippo Neri Community Hospital of Rome between January 2017 and December 2023.

We divided the patients into two groups: group A, comprising 102 patients who underwent surgery between January 2017 and February 2020 and were operated on without FS, and group B, comprising 55 patients who underwent surgery between February 2020 and December 2023 and were operated on with the use of FSAS.

All patients underwent magnetic resonance imaging (MRI) scans, including both non-contrast and gadolinium-contrast imaging, within 1 month prior to admission. Tumor measurements were taken in three dimensions, based on the axial and coronal MRI section planes, and the maximum diameter in centimeters was estimated. The tumors were then classified according to the Koos et al. [13] classification system.

The extent of surgical removal was classified as follows: total (100%), near-total (>95%), subtotal (95–90%), and partial (<90%). The extent of removal was evaluated intraoperatively and using postoperative gadolinium-enhanced MRI, which was performed 24–48 h after surgery.

Facial nerve (N VII) function was evaluated preoperatively using the House-Brackmann (HB) grading system [14]. Additionally, facial nerve function was assessed immediately postoperatively and 6 months after follow-up.

The preoperative audio-vestibular evaluation included pure-tone audiometry and speech audiometry. The hearing levels were graded according to the American Academy of Otolaryngology-Head and Neck Surgery (AAO-HNS) classification system [15]. Classes A and B were considered socially useful hearing, while hearing preservation corresponded to classes A, B, and C, with class C indicating a level of hearing less than 50% that was not socially useful. Hearing preservation was attempted only in patients who had a preoperative hearing level classified as A or B.

### 2.2. Surgical Technique

All operations (for both groups) were performed using the retrosigmoid approach in the lateral Fukushima position with the use of intraoperative neuromonitoring. Neuronavigation was not used in any case (the retrosigmoid approach was performed with the use of anatomical landmarks starting with skin incision—the superior and inferior nuchal lines, then craniotomy—the digastric groove, asterion, and the opening of the internal auditory canal—the suprameatal tubercle and Tübingen line, and the tumor was debulked from inside until the capsule became liable to move and remove).

All patients in group B received FS at a standard dose of 5 mg/kg at the time of skin incision. All operations were performed using a surgical microscope with an integrated fluorescein filter, specifically the Leica ARveo ^®^ FL560 model (Leica Microsystems, Wetzar and Mannheim, Germany), which enabled the observation of fluorophores with an excitation range of 460–500 nm. This allowed for the simultaneous viewing of non-fluorescent tissue in a natural color and fluorescent tissue in a bright yellowish-green color.

### 2.3. Clinical Follow-Up

Clinical and radiological follow-ups were scheduled for six months after the operation and then annually, with the final date set for 15 December 2023.

The facial nerve outcomes were categorized according to the House-Brackmann grading system, which ranges from grade I to grade VI. The hearing level was classified according to the American Academy of Otolaryngology-Head and Neck Surgery (AAO-HNS) classification system, which ranges from A to D. Notably, there were no patients lost to follow-up. However, ten patients underwent surgery less than six months prior to the scheduled follow-up time and, therefore, did not meet the required follow-up period. To address this issue, missing data values were substituted with median values that had been calculated for each variable.

### 2.4. Statistical Analysis

The statistical analysis was conducted using SPSS software (version 25), and *p*-values < 0.05 were considered significant, while *p*-values ≥ 0.05 were considered non-significant (NS).

The use of median imputation to eliminate and handle missing data allowed for a robust study, thereby avoiding any bias in interpreting the findings. All statistical tests and analyses were performed on the imputed dataset to ensure that all findings were valid.

## 3. Illustrative Cases

### 3.1. Case Number 1

**Figure 1:**
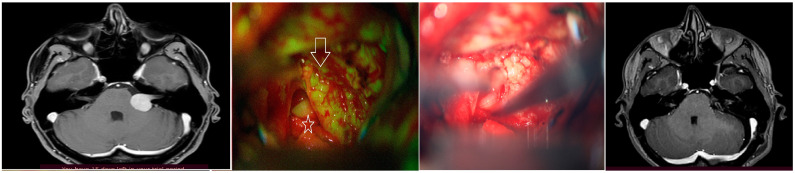
This figure illustrates a preoperative MRI with contrast, as well as intraoperative views of the tumor, which is depicted in yellow-green color under the filter (indicated by the arrow). The facial nerve is white (star). The figure also includes a normal microscopic view without a filter and a postoperative control MRI for comparison.

A 34-year-old female patient, previously unremarkable in her medical and surgical history, presented with symptoms of ataxia and hearing impairment. Upon physical examination, the patient’s condition was found to be intact, except for the ataxia. MRI revealed a left-sided VS, classified as Koos grade 3. Based on the patient’s condition, surgical excision was planned and performed with the intraoperative assistance of FS, as depicted in Figure 1. Notably, under the microscope filter, the tumor was observed to be yellowish-green in color, while the facial nerve remained normal in its white color. Additionally, a postoperative MRI control was performed within 24–48 h to assess the patient’s outcome.

### 3.2. Case Number 2

**Figure 2:**
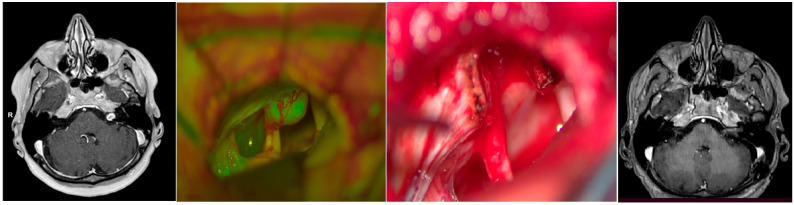
This figure illustrates an MRI study of a small VS as well as intraoperative imaging with a yellow 560 filter. The image shows the tumor arising from the internal auditory canal, enhancing yellowish-green color, and the non affected nerve appears white. The image on the right depicts the final result after opening the canal and removing the tumor, and the postoperative MRI control images are also included.

A 60-year-old female patient with a history of hypertension presented with a 5-year decline in hearing due to a small VS. Despite her initial hearing loss, the VS exhibited further growth over the past year with further hearing impairment. Initially, the patient refused radiation therapy and instead opted for observation and potential surgical intervention. After being presented with options for management (observation, radiotherapy, and surgical excision), she elected to undergo surgical excision. The patient’s preoperative, intraoperative, and postoperative images are displayed in Figure 2. Postoperatively, she experienced HB grade 2 facial weakness, which resolved after one week. At three-month follow-up, her facial nerve function had returned to normal.

### 3.3. Case Number 3

In the Supplementary Materials you can find a short intraoperative video for FS-assisted surgery case.

## 4. Results

The total number of patients included in this study was 157 patients.

This study comprised two groups: group A, comprising 102 patients (63 females and 39 males) with no intraoperative FS use, including 56 patients who had tumors on the right side, and the mean tumor size was 2.64 cm. Group B, comprising 55 patients (32 females and 23 males), received intraoperative FS, including 29 patients who had tumors on the right side, and the mean tumor size was 2.34 cm. The observed side effects of FS were one case of a moderate reaction (extravasation and skin eruption), which was 1.8%, and two cases of mild reactions (nausea and vomiting), which was 3.6%.

The majority of patients in both cohorts with and without intraoperative FS rated their Koos grade as 2, with frequencies of 36.3% and 49.1%, respectively.

Regarding the extent of removal, most patients who did not receive intraoperative FS underwent complete resections in 74.5% of cases, followed by near-total resections in 19.6% of cases, while only a small percentage (5.9%) had subtotal resections.

The total resection rate was 56.4% in patients treated with FSAS, while near-total resections accounted for 40% of cases, resulting in a subtotal resection rate of 3.6%.

Table 1 and Table 2 provide a summary of the clinical and radiological findings for each group.

A crosstabulation analysis was conducted to examine the relationship between the preoperative hearing loss (PRE-HL) assessment and postoperative hearing loss (POST-HL) assessment, as presented in Table 3 and Table 4.

To investigate whether there was a difference in the tumor resection degree between the patients who received FS and those who did not, we performed a Mann–Whitney test. The resulting *p*-value of 0.037 indicated statistical significance, suggesting a significant difference in the extent of the removal rates between the two groups, as shown in Table 5. Specifically, the mean rank for patients receiving fluorescein was 87.42, compared to 74.42 for those who did not receive fluorescein. The Z-value of −2.09, which reflects a mild observed difference in accordance with Cohen’s 1992 standards for effect size interpretation, suggests that this difference is considered effective.

After finding a statistically significant correlation between sodium fluorescein administration and tumor extension excision, we performed further analyses by categorizing patients according to the Koos classification. We then conducted separate Mann–Whitney tests within each Koos category to examine the specific effect of fluorescein on different clinical stages. Notably, we obtained a *p*-value of 0.001 for the Koos 3 group, indicating that fluorescein administration had a significant effect on this specific subgroup. The mean rank results showed that patients who received fluorescein had a mean rank of 33.7, compared to 21.9 for those who did not receive fluorescein.

Additionally, we used the Mann–Whitney test to investigate the effect of fluorescein administration on hearing loss and facial nerve function. The results showed no statistically significant difference between the patients who received fluorescein and those who did not in terms of either hearing loss or facial nerve function. The *p*-values for all three outcomes were non-significant, exceeding the 0.05 significance threshold, indicating a lack of significance, as shown in Table 5.

A Spearman’s correlation analysis was employed to examine the relationship between tumor removal extension and various parameters, including Koos, age, tumor size, postoperative assessment of hearing loss, and early and late assessments of facial nerve function, as presented in Table 6.

## 5. Discussion

### 5.1. Management of Vestibular Schwannoma

VS treatment options are diverse and may include observation, surgical resection, and radiation therapy. Each option is tailored to individual patient circumstances, considering factors such as age, overall health, tumor size, symptoms, growth rate, and other relevant characteristics. Treatment options can be adjusted as needed based on changes in patient status, such as symptom progression or growth rate. Surgical resection techniques are several, which may include retrosigmoid, translabyrinthine, subtemporal approaches, or combinations of these approaches, depending on factors such as tumor size, surgeon expertise, and intracanalicular extension.

Resection surgeries aiming for complete resection have been shown to have the most beneficial outcomes, with significantly better short-term and long-term results compared to incomplete tumor removal. Notably, patients who undergo complete resection experience lower rates of recurrence and higher rates of successful resection [16]. A comprehensive review of 1000 VS resections reported a remarkable 98% complete resection rate, as well as a 68% hearing preservation rate. Additionally, the mortality rate associated with these surgeries was remarkably low, at just 1% [17]. Notably, the risk of local recurrence after a complete resection is extremely low, ranging from 0% to 2%. In contrast, if only the partial removal of the tumor is possible, the incidence of tumor recurrence is significantly higher, at approximately 30% [17]. Despite advances in surgical techniques, the complete resection of VS may still be challenging due to their adhesion to surrounding nerves or brain structures, which can increase the risk of incomplete resection or damage to normal tissues [18]. This uncertainty has led to questions about the potential benefits of integrating neural-guided techniques into intracranial tumor resection, which could potentially improve gross total resection while protecting normal vital tissues and enhancing patient outcomes.

### 5.2. Application of Fluorescein Sodium

The first application of fluoresceine in neurosurgery was in 1948 by Moore et al. [19], where it was used for the identification of several types of brain tumors, and recently it has been used more in high-grade gliomas [20], meningiomas [21], skull base tumors [10], less common VS [12], and peripheral schwannomas [22].

### 5.3. Safety of Fluorescein Sodium

FS is now widely used in neurosurgery for oncology and neurovascular purposes. It has been applied for many years in general surgery, gastroenterology, and mainly in ophthalmology with a safe profile [23].

Yannuzzi et al. [23] classified the adverse reactions of the intravenous use of FS based on the duration, need for intervention, and final outcome into mild, moderate, or severe reactions. The severe reactions—including cardiovascular, respiratory, neurological, or death—were extremely rarely reported, and in our series, we did not report any severe reactions. Moderate reactions included the development of skin eruption, syncope, local tissue necrosis, thrombophlebitis, pyrexia, and nerve palsy. The development of skin eruption represented the most common of the moderate reactions, and we had one case that developed skin eruption that was treated with a regular dressing and oral antihistamine. Mild reactions represented the most common reactions to FS, represented by nausea and vomiting, extravasation, inadvertent intraarterial injection, sneezing, and pruritis. In our series, we had two cases that developed nausea and vomiting; it was for one to two days and then resolved spontaneously, and another case that had an extravasation, which was treated conservatively with arm raises and exercise.

Restelli et al. [24] conducted a comprehensive neurosurgical literature review on the safety of FS and found that FS was shown to be extremely safe in neurosurgery, including oncological and neurovascular cases, even at high doses.

### 5.4. Dosage of Fluorescein Sodium

FS has been used in neurosurgery at various stages, where it was previously used at large dosages (40 mg/kg) for a low-grade glioma or a re-dose was used to promote tumor enhancement under a microscope, with the main reason being a lack of fitted specific filters on surgical microscopes [20,25,26]. According to recent extensive research [24], the current tendency is to utilize lower doses (about 5 mg/kg) due to the availability of microscopes with wavelength-specific filters for FS. In our series, we employed a fixed dosage and timing of 5 mg/kg at the time of skin incision.

### 5.5. Mechanism of Enhancement

Yan Xiang et al. [27] found that sodium fluoresceine-assisted surgery is effective for high-grade glioma surgery since FS presence is directly related to the breakdown of the blood-brain barrier (BBB) in the tumor. On the counterpart, VSs are benign and often hypervascular tumors, and the presence of FS could be explained by the pathological blood vessels of the tumor rather than the breakdown of the BBB. Therefore, FS can be used to aid in the recognition of the facial and cochlear nerves from the tumor, potentially assisting the surgeon in anatomical preservation for the normal nerves with a greater extent of resection, as we found from our comparison results.

### 5.6. Maximal Safe Resection

The existing literature consistently highlights a notable trend: incomplete resection for patients with VS is linked to a higher incidence of progression or regrowth compared to patients who underwent total resection [28]. Based on the current knowledge, the present study researched a narrow spectrum, studying the possibility of sodium fluorescein’s impact on VS resection and follow-up clinical outcomes. Our goal was to focus on this particular aspect and create a comprehensive discussion on the greater completeness of tumor removal, which may increase the various gain rates in the administration of FS in patients with VS. This specific research is in line with the final objective, which is to improve treatment options and enhance the outcomes of patients in the context of VS management.

Fluorescein-guided surgery allows for the more precise identification of tumor borderlines from the adjacent structures (the facial nerve, trigeminal nerve, lower cranial nerves, and brainstem), simplifying a more radical resection. The data of our study shows a significant difference in tumor removal between the patients who received fluorescein and those who did not, with the greatest differences observed among the patients categorized as Koos 3. As a result, the operation may have been performed more radically using fluorescein to be sure of the complete removal of the tumor while the important structures are preserved. The results endorse the use of fluorescein as a targeted adjunct in VS microsurgery, which in turn opens the door for the further development of surgical techniques.

### 5.7. Hearing Level

The positive and significant correlation between tumor resection and hearing loss in patients undergoing surgery is also of particular interest. This implies that more extensive tumor removal comes at the cost of more destruction of the auditory structures, thus producing worse hearing outcomes. This might happen due to the close adhesion of the tumor to the vital auditory structures like the cochlear nerve during operation [29,30]. Other studies have suggested that the surgical technique can preserve hearing function, especially in smaller tumors [30,31].

### 5.8. Facial Nerve

The results of our research demonstrate no statistically significant correlation between the extent of tumor resection and facial nerve function. This is consistent with findings from many other studies [32,33]. This finding indicates that the use of fluorescein does not lead to improved facial motor function outcomes, especially when looked at from the perspective of VS removal. Other factors that may turn out to be an important determinant of facial nerve function outcomes include the tumor size, adherence of the tumor to the nerve, hypervascularized tumors, facial nerve function before the operation, and age of the patient [32,33,34].

### 5.9. Limitations

Although this study has provided us with some invaluable knowledge, some limitations have to be noted. The limitations associated with this study may include the sample size of a known cohort and the nature of a retrospective analysis, which may bring about some biases. Future studies should be directed at filling these gaps since fluorescein can only be considered as having the potential to cause changes in the tumor resection results, and this may have a clinical impact.

### 5.10. Summary

Our cohort study provides valuable insights into the role of sodium fluorescein in VS surgery, particularly regarding its impact on the extent of resection and postoperative functional outcomes for facial and cochlear nerves. Notably, FS was found to be safe and well-tolerated at a dose of 5 mg/kg, with only mild to moderate adverse reactions reported. However, further research is needed to fully assess the safety of FS in neurosurgery practice, including the optimal timing and dosing, as well as the possibility of redosing if necessary.

## 6. Conclusions

FS-assisted surgery for VS may have a substantial influence on the extent of tumor resection, with no discernible effects on postoperative hearing and facial nerve function. Moreover, FS is deemed safe for use in VS surgery.

## Figures and Tables

**Table 1:** Analysis of age and tumor size for both groups.

Fluoresceine		Age	Size
No	Mean	50.12	2.642
	Number	102	102
	Standard Deviation	13.522	0.935
Yes	Mean	51.36	2.340
	Number	55	55
	Standard Deviation	10.94	0.837
Total	Mean	50.55	2.536
	Number	157	157
	Standard Deviation	12.655	0.911

**Table 2:** Clinical and radiological characteristics for both groups in this study.

Fluoresceine		Sex		Koos				Removal Extent		
		M	F	1	2	3	4	T	NT	ST
No	Frequency	39	63	1	37	35	29	76	20	6
	Percent	38.2	61.8	1	36.3	34.3	28.4	74.5	19.6	5.9
Yes	Frequency	23	32	3	27	15	10	31	22	2
	Percent	41.8	58.2	5.5	49.1	27.3	18.2	56.4	40	3.6

T: total resection, NT: near-total resection, ST: subtotal resection.

**Table 3:** Fluoresceine PRE-HL crosstabulation count.

		PRE-HL				Total
		A	B	C	D	
Fluoresceine	No	7	21	47	27	102
	Yes	1	20	24	10	55
Total		8	41	71	37	157

**Table 4:** Fluoresceine POST-HL crosstabulation count.

		POST-HL					Total
		A	B	C	Missing	D	
Fluoresceine	No	4	11	33	0	54	102
	Yes	1	7	21	10	16	55
Total		5	18	54	10	70	157

**Table 5:** Mann–Whitney test.

	Removal Extent	Early HB	Post HL	Late HB
Mann–Whitney U	2342.000	2623.000	2410.000	2668.500
Wilcoxon W	7595.000	4163.000	3950.000	7921.500
Z	−2.090	−0.797	−1.559	−0.765
Asymp. Sig. (2-tailed)	**0.037**	0.425	0.119	0.444

Significant differences in tumor resection rates were observed between the patients with and without fluorescein (*p* = 0.037), However, fluorescein showed no significant impact on hearing loss or facial nerve function, with all *p*-values exceeding 0.05.

**Table 6:** Results of the Spearman’s correlation analysis.

		Koos	Age	Size	Post HL	Late HB	Early HB
Removal	Correlation Coefficient	0.270	0.167	0.306	0.230	0.123	0.109
	Sig. (2-tailed)	0.001	0.037	0.000	0.004	0.126	0.175
	Number	157	157	157	157	157	157

Positive correlations between the degree of extensions to Koos, age, size, and hearing loss assessment after surgical procedures. No statistically significant correlation was found between the early and late assessments of facial nerve function.

## Data Availability

The data are only available upon request from the last author due to restrictions, privacy, legal, or ethical reasons.

## Supplementary Materials

The following supporting information can be downloaded at: https://www.mdpi.com/article/10.3390/brainsci14060571/s1. This video shows a patient with a left VS, classified as Koos 3, undergoing surgery using intraoperative neuromonitoring in the lateral Fukushima position via the retrosigmoid approach. The surgical approach begins by carefully checking the facial nerve position, followed by opening of the capsule using a hand-held Thulium laser. Next, the tumor is debulked intracapsular to make it suitable for detachment from surrounding tissue. As part of the procedure, the facial nerve is identified and verified using a filtered lens (FL560), which allows us to visualize the nerve in its normal white color and distinguish it from the tumor. The tumor is then glowing yellowish-green, while the capsule is dark and non-enhancing. This allows for careful dissection of the tumor from the nerve, minimizing potential injury or traction. Finally, postoperative control MRI is shown at the end of the video to confirm successful tumor resection. This video demonstrates a successful application of fluorescein-assisted surgery for VS resection, highlighting the potential benefits of this technique for improved patient outcomes.
